# Comparative Proteomic Analysis of the Defense Response to *Gibberella* Stalk Rot in Maize and Reveals That ZmWRKY83 Is Involved in Plant Disease Resistance

**DOI:** 10.3389/fpls.2021.694973

**Published:** 2021-08-13

**Authors:** Hua Bai, Helong Si, Jinping Zang, Xi Pang, Lu Yu, Hongzhe Cao, Jihong Xing, Kang Zhang, Jingao Dong

**Affiliations:** ^1^State Key Laboratory of North China Crop Improvement and Regulation, Hebei Agricultural University, Baoding, China; ^2^Hebei Key Laboratory of Plant Physiology and Molecular Pathology, Hebei Agricultural University, Baoding, China; ^3^College of Life Sciences, Hebei Agricultural University, Baoding, China; ^4^College of Plant Protection, Hebei Agricultural University, Baoding, China

**Keywords:** maize, proteomic, stalk rot, *Fusarium graminearum*, ZmWRKY83

## Abstract

*Fusarium graminearum* is the causal agent of *Gibberella* stalk rot in maize stem, resulting in maize lodging, yield, quality, and mechanical harvesting capacity. To date, little is known about the maize stem defense mechanism in response to the invasion of *F. graminearum*. This study represents a global proteomic approach to document the infection by *F. graminearum*. A total of 1,894 differentially expressed proteins (DEPs) were identified in maize stem with *F. graminearum* inoculation. Functional categorization analysis indicated that proteins involved in plant-pathogen interaction were inducible at the early stages of infection. We also found that the expression of proteins involved in phenylpropanoid, flavonoid, and terpenoid biosynthesis were upregulated in response to *F. graminearum* infection, which may reflect that these secondary metabolism pathways were important in the protection against the fungal attack in maize stem. In continuously upregulated proteins after *F. graminearum* infection, we identified a WRKY transcription factor, ZmWRKY83, which could improve the resistance to plant pathogens. Together, the results show that the defense response of corn stalks against *F. graminearum* infection was multifaceted, involving the induction of proteins from various immune-related pathways, which had a directive significance for molecular genetic breeding of maize disease-resistant varieties.

## Introduction

Plants are stressed by various pathogens in nature. However, few pathogens can be successfully colonized in host plants, which indicates the existence of recognition and defense mechanisms. Plants have evolved multiple defense strategies for combating invading pathogens. Pathogen-associated molecular patterns (PAMPs)-triggered immunity (PTI) and effector-triggered immunity (ETI) are well-defined modes of plant immunity against pathogens (Thomma et al., 2011). Infected organisms recognize that they are under attack by detecting pathogens directly through PAMPs that bind to pattern recognition receptors. The infected host can identify pathogens by ETI pattern recognition, thereby alerting the host to pathogens through associated damage caused by pathogenic toxins or effectors (Akira et al., 2006; Kazan and Lyons, 2014; Cui et al., 2015). Reactive oxygen species production, MAP kinase activation, which is related to immune responses, can occur in both PTI and ETI (Tsuda and Katagiri, 2010).

Maize is one of the most important crops worldwide. Unfortunately, maize is susceptible to a variety of pathogens during growth and development. Among the fungal pathogens, *Fusarium graminearum* is the causative agent of ear and stalk rot disease in maize (Santiago et al., 2007). In maize, when the stalks die prematurely, the plant produces light ears with poor grain filling, or when the stalks fall, it causes significant losses and leads to failure in the harvesting of ears (Mesterhazy et al., 2012; Gai et al., 2018). However, the disease resistance mechanisms during maize stalk infection with *F. graminearum* are not clear, which limits progress in effective disease control. Several quantitative trait loci for resistance have been reported (Pe et al., 1993; Yang et al., 2010; Zhang et al., 2012; Chen et al., 2017; Ma et al., 2017). *ZmAuxRP1*, which encodes a plastid stroma-localized auxin-regulated protein, is demonstrated as a key gene at *qRfg2* through map-based cloning (Ye et al., 2018). *ZmPRms* has been speculated to be involved in resistance to infection by *Aspergillus flavus* and other pathogens (Majumdar et al., 2017b).

Proteins, as direct executors, participate in plant growth and development, secondary metabolism, and many other processes, reflecting the response of various physiological functions to biotic or abiotic stress stimuli (Hu et al., 2015; Yue et al., 2018; Zhang et al., 2019). Quantitative proteomics focuses on screening and identifying proteome variations among different species or states, revealing, and verifying changes in proteomics (Dunkley et al., 2006; Pechanova et al., 2013). Several studies have used proteomics to explore the response to biotic stress in maize (Huang et al., 2009; Pechanova et al., 2013). Comparative proteomics analysis of maize rachis protein explores resistance and susceptibility to aflatoxin accumulation (Pechanova et al., 2011). Proteome changes induced by *F. graminearum* inoculation for 48 h were evaluated in developing grains of two inbred lines (Mohammadi et al., 2011). To verify the hypothesis that the primary metabolism of *Azospirillum brasilense* Sp7 is different from that of C3 and C4 plants, differential proteomics was used to study the protein expression in maize and tomato with *A. brasilense* treatment (Lade et al., 2018). Proteomics data proved that effector HC toxin is a histone deacetylase inhibitor produced by fungal pathogen *Cochliobolus carbonum* race 1, which promotes maize virulence by altering protein acetylation (Walley et al., 2018). Comparative proteomics analysis was employed to select candidate proteins related to southern corn rust resistance in susceptible and resistant maize lines (Wang et al., 2019).

Transcription factors play a key role in crosstalk regulation between multiple hormones signaling pathways and signal-transduction-mediated defense gene expression. The WRKY transcription factors form a highly interconnected regulatory subnetwork in *Arabidopsis* immune response (Rushton et al., 2010; Llorca et al., 2014). WRKY33 is a key regulator in defense against *Botrytis cinerea* from the WRKY transcription factor family (Liu et al., 2017). At the early stages of *B. cinerea* infection, the expression level of *WRKY33* is highly induced (Birkenbihl et al., 2017b; Sham et al., 2017). In the *WRKY33* mutant, knock out of WRKY33 increases sensitivity of plants to *B. cinerea*, whereas overexpression of WRKY33 reduces susceptibility to *B. cinerea* (Zheng et al., 2006; Birkenbihl et al., 2012). After pathogen infection, WRKY33 can activate defense response mechanisms *via* MAP-dependent phosphorylation (Wang et al., 2018b; Han et al., 2019). In addition, WRKY33 homologous proteins can regulate plant disease resistance is confirmed in other plants, such as *Solanum lycopersicum*, *Vitis vinifera*, and *Brassica napus* (Wang et al., 2014; Merz et al., 2015; Zhou et al., 2015). Therefore, it is necessary to fully understand the plant defense mechanisms mediated by WRKY33 and its homologous proteins.

Maize stalk rot, caused by members of the *F. graminearum* species complex, is among the most destructive and economically important diseases in the world (Yang et al., 2010). However, very little is known about the defense responses against *F. gramincarum* infection in maize stem. In this study, we used high-throughput tandem mass tag (TMT)-based technology for proteomics comparison to explore pathogen-responsive proteins and biological processes in maize stems, which showed moderate resistance to *F. gramincarum* infection. We identified several defense-related proteins and revealed several secondary metabolism pathways associated with the defense responses. In addition, we also verified that ZmWRKY83 was a key transcription factor in plant disease resistance. This study not only provided several important information to understand the molecular mechanism of the interaction between maize and *F. graminearum* but also provided important clues for the genetic breeding of disease-resistant maize varieties.

## Results

### Global Proteomic Changes With *F. graminearum* Infection in Maize

**FIGURE 1:**
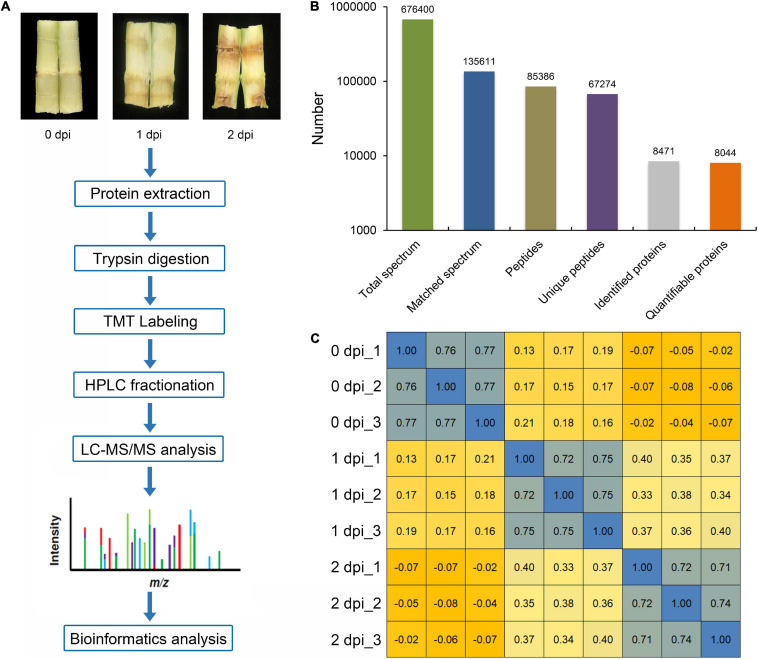
Overview of treatments and proteome profiling. **(A)** Differentially expressed proteins (DEPs) were quantified and analyzed by tandem mass tag (TMT) labeling and LC-MS/MS. **(B)** Summary of sampled spectra, peptides, acetylated peptides, and identified proteins. **(C)** Pearson correlation statistics of the replicates of every experiment.

**FIGURE 2:**
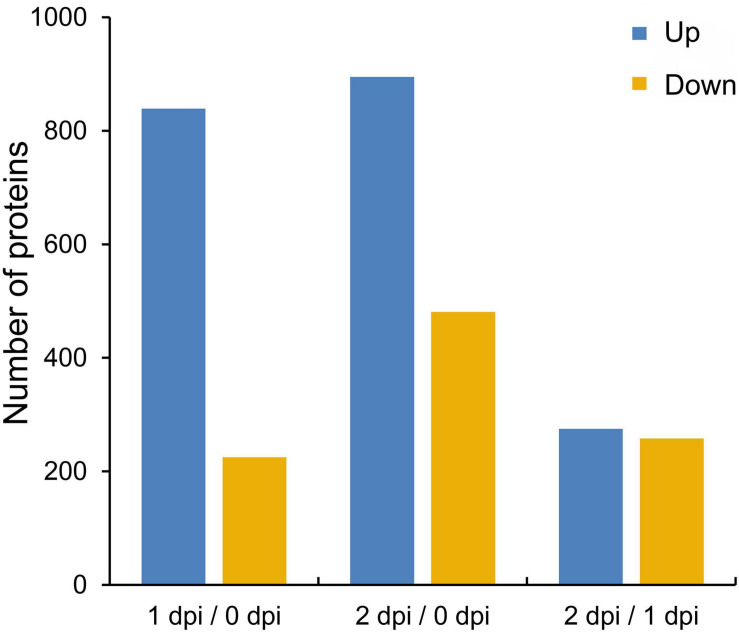
The numbers of upregulated and downregulated proteins in maize stem following *Fusarium graminearum* infection. A cutoff of 1.3-fold and *p*-value < 0.05 were used to select DEPs.

*Fusarium graminearum* can cause very serious stalk rot disease in maize. Therefore, it was important to explore the resistance mechanism of stalk rot caused by *F. graminearum* in maize. To investigate the global proteomic changes in response to *F. graminearum* infection in maize stem, comparative proteomics-based TMT labeling technology was conducted with three independent biological replicates for each sample (Figure 1A). A total of 676,400 spectra were generated from TMT analysis, after searching the maize sequence database, yielding 85,386 matched peptides, 67,274 unique peptides, 8,471 matched proteins, and 8,044 quantified proteins (Figure 1B and Supplementary Table 1), and the biological replications in different infection stage showed good similarity (Figure 1C and Supplementary Figure 1). According to a criterion of 95% significance and a 1.3-fold cutoff (*p*-value < 0.05), 1,894 proteins were identified as differentially expressed proteins (DEPs) between 0, 1, and 2 days postinoculation (dpi; Supplementary Table 2). Then, we identified 1,064 DEPs in 1 dpi and 1,376 DEPs in 2 dpi that were significantly up or down expressed compared with 0 dpi (Figure 2). Among the 1,894 DEPs, we found 100 proteins were continuously upregulated (Supplementary Figure 2A), while 14 proteins were continuously downregulated after *F. graminearum* infection (Supplementary Figure 2B).

### Function Analysis of DEPs Identified by Proteomics

**FIGURE 3:**
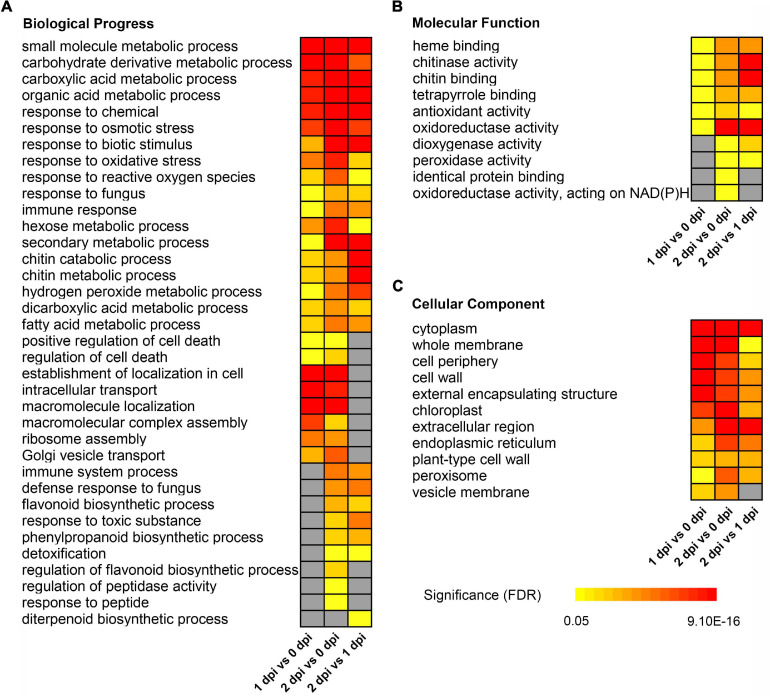
Gene ontology enrichment of up-regulated differentially expressed proteins between 0, 1, and 2 dpi. Enriched GO terms showed in biological progress **(A)**, molecular function **(B)**, and cellular component **(C)**. **(C)** The colors from yellow to red represent the significance level of the GO terms from high to low. Fisher’s exact test, *P*-value was adjusted using the Benjamini-Yekutieli method, FDR < 0.05.

To further understand the identified and quantified functions and characteristics of proteins, we conducted GO enrichment analysis for these DEPs in response to *F. graminearum* infection. For upregulated DEPs, the GO terms “small molecule metabolic process” [GO:0044281, false discovery rate (FDR) < 8.30E-15], “response to biotic stimulus” [GO:0009607, FDR < 8.40E-5], “chitin catabolic process” [GO:0006032, FDR < 0.0026], “hydrogen peroxide metabolic process” [GO:0042743, FDR < 0.0085] in biological process (Figure 3A), “heme binding” [GO:0020037, FDR < 0.0025], “chitinase activity” [GO:0004568, FDR < 0.0058], “oxidoreductase activity” [GO:16491, FDR < 0.0095] in molecular function (Figure 3B), etc., were significantly enriched throughout the infection stage. In addition, some GO terms were significantly enriched from 1dpi to 2 dpi, such as “flavonoid biosynthetic process” [GO:0009813, FDR < 0.0027], “response to toxic substance” [GO:0009636, FDR < 0.00091], “phenylpropanoid biosynthetic process” [GO:0009699, FDR < 0.0018], “dioxygenase activity” [GO:0051213, FDR < 0.018] (Figures 3A,B), etc. GO terms in cellular component were significantly enriched cytoplasm [GO:0005737, FDR < 5.50E-14], whole membrane [GO:0098805, FDR < 0.0049], and extracellular region [GO:0005576, FDR < 7.20E-06] (Figure 3C), etc.

**FIGURE 4:**
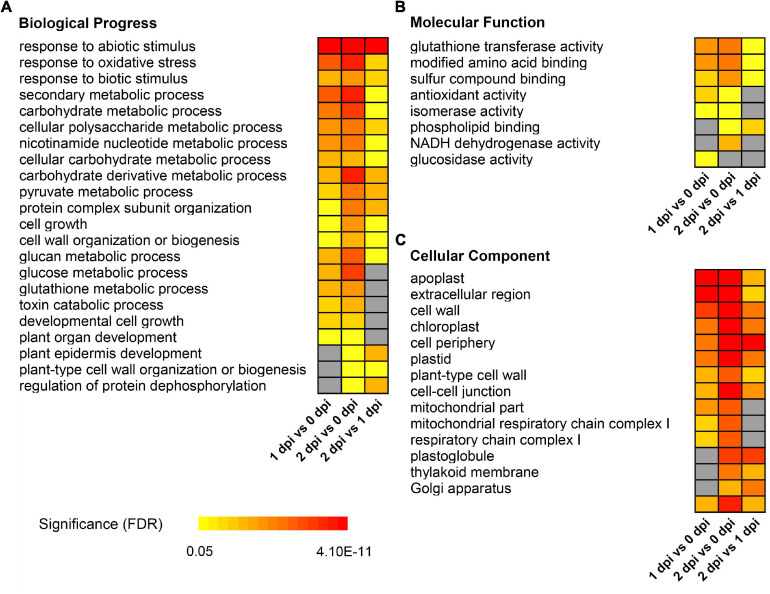
Gene ontology enrichment of down-regulated differentially expressed proteins between 0, 1, and 2 dpi. Enriched GO terms showed in biological progress **(A)**, molecular function **(B)**, and cellular component **(C)**. The colors from yellow to red represent the significance level of the GO terms from low to high. Fisher’s exact test, *P*-value was adjusted using the Benjamini-Yekutieli method, FDR < 0.05.

For downregulated DEPs, the carbohydrate metabolism related GO terms were significantly enriched, such as “carbohydrate metabolic process” [GO:0005975, FDR < 0.001], “cellular polysaccharide metabolic process” [GO:0044264, FDR < 0.0015], and “glucan metabolic process” [GO:0044042, FDR < 0.0056], etc (Figure 4A). Meanwhile, GO terms related to plant growth and development, such as “cell growth” [GO:0016049, FDR < 0.013], “cell wall organization or biogenesis” [GO:0071554, FDR < 0.0015], “plant-type cell wall” [GO:0009505, FDR < 0.00082], etc., were also significantly enriched (Figures 4A,C). Moreover, “protein complex subunit organization” [GO:0071554, FDR < 0.0015], “regulation of protein dephosphorylation” [GO:0071554, FDR < 0.0015], “glutathione transferase activity” [GO:0009505, FDR < 0.00082], etc., were also enriched in downregulated DEPs (Figures 4A,B). These results suggested that maize might modulate the immune system in response to the *F. graminearum* infection by slowing down plant growth and energy metabolism.

### The Kyoto Encyclopedia of Genes and Genomes (KEGG) Enrichment Analysis of DEPs

**FIGURE 5:**
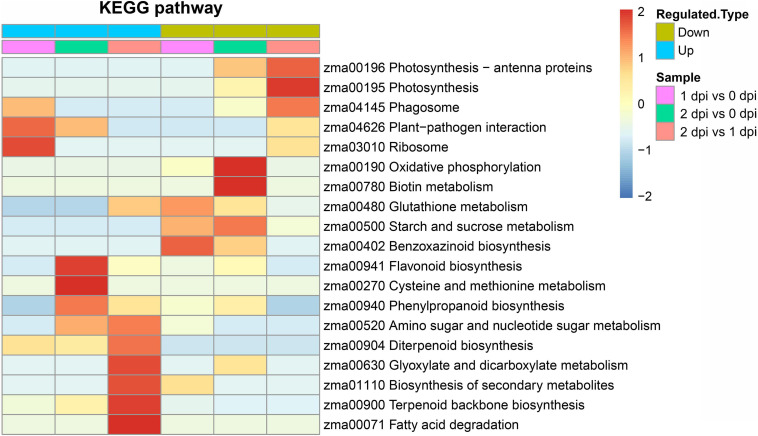
Kyoto encyclopedia of genes and genomes (KEGG) enrichment analysis of upregulated and downregulated DEPs between 0, 1, and 2 dpi. The colors from blue-to-red represent the significance level of the KEGG pathways from low to high.

To further analyze the key enriched metabolic pathways in response to *F. graminearum* infection, The KEGG pathway enrichment analysis was performed with pathogen-responsive proteins from the respective proteomes. The KEGG analysis demonstrated that phagosome, plant-pathogen interaction, and ribosome were significantly affected and upregulated by the early stage of *F. graminearum* infection (1 dpi). Compared with 2 dpi proteomic data, flavonoid biosynthesis, phenylpropanoid biosynthesis, amino sugar, and nucleotide sugar metabolism, diterpenoid biosynthesis, glyoxylate, and dicarboxylate mentalism, biosynthesis of secondary metabolites, terpenoid backbone biosynthesis, and fatty acid degradation were significantly enriched in upregulated DEPs (Figure 5). These results suggested that the disease resistance genes could mediate plant-pathogen interaction and the synthesis and metabolism of secondary metabolites in response to pathogen infection.

The KEGG pathway includes glutathione metabolism, starch and sucrose metabolism, oxidative phosphorylation, biotin metabolism, and photosynthesis that were enriched in downregulated DEPs (Figure 5), which suggested that energy metabolism and plant development were downregulated to resist *F. graminearum* infection. In addition, the results of KEGG analysis were relatively consistent with GO enrichment, and all of these results might reflect how plants resist fungal infection.

### Transcriptional Expression Pattern of Some DEPs in Response to *F. graminearum* Infection

**FIGURE 6:**
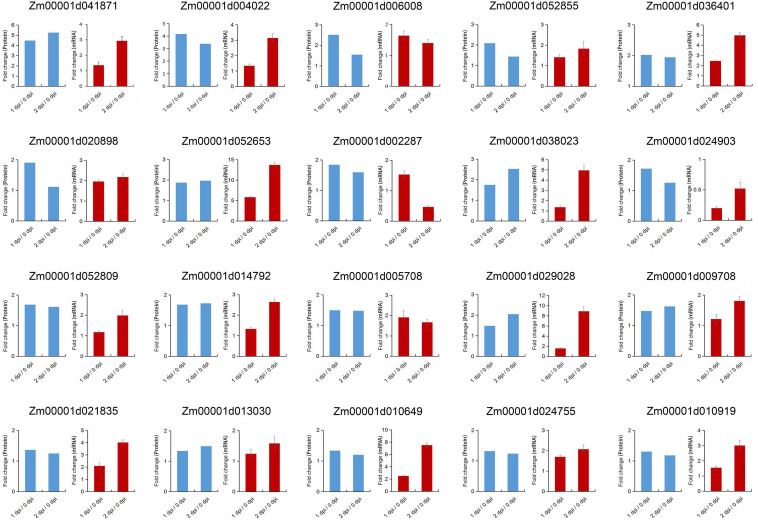
Correlation between proteomics and quantitative real-time PCR (qRT-PCR) results of the proteins enriched in plant-pathogen interaction pathway. Error bars represent SD from the mean.

**FIGURE 7:**
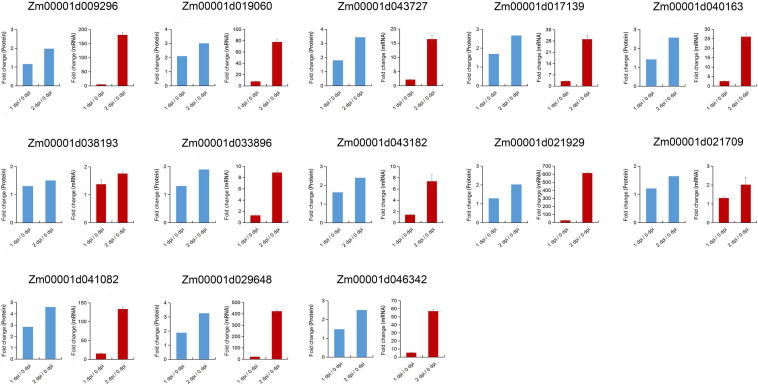
Correlation between proteomics and qRT-PCR results of the proteins enriched in terpenoid backbone and diterpenoid biosynthesis pathway. Error bars represent SD from the mean.

In KEGG pathway enrichment analysis, plant-pathogen interaction was significantly enriched in the upregulated DEPs with *F. graminearum* early infection, while terpenoid backbone and diterpenoid biosynthesis were enriched in later infection (Figure 5). To further examine relationships between mRNA expression and protein abundance under *F. graminearum* infection, the relative transcript level of proteins enriched in plant-pathogen interaction, terpenoid backbone, and diterpenoid biosynthesis was examined by quantitative real-time PCR (qRT-PCR). Transcript levels of these proteins (excepted Zm00001d024903) enriched in plant-pathogen interaction were successfully determined and mostly increased in both 1 and 2 dpi, compared with 0 dpi (Figure 6). The proteins enriched in terpenoid backbone and diterpenoid biosynthesis were also successfully determined. Except for Zm00001d038193 and Zm00001d021709, most of them were observably upregulated during *F. graminearum* infection, especially in 2 dpi (Figure 7). In addition, we also examined the transcript levels of proteins enriched in the benzoxazinoid biosynthesis pathway which were downregulated, and all transcript levels of these proteins were decreased by *F. graminearum* infection.

Then, we compared the expression level of the genes enriched in plant-pathogen interaction, terpenoid backbone, and diterpenoid biosynthesis pathway in susceptible (B73) and resistant (Mo17) lines. We found that the expression levels of these genes were also significantly upregulated in Mo17 (Supplementary Table 4). However, most of the genes in Mo17 plants had higher multiples of expression than in B73, especially in 1 dpi. Moreover, we further extend the expression analysis in 0, 1, 2, and 3 dpi (Supplementary Table 5). Compared with 0 dpi, qRT-PCR results showed that most genes enriched in plant-pathogen interaction, terpenoid backbone, and diterpenoid biosynthesis pathways were significantly upregulated in 3 dpi, whereas increase multiple was not as high as 2 dpi. These results indicated that these genes played important roles in disease resistance.

### ZmWRKY83 Was a Key Transcription Factor in Disease Resistance

In the plant-pathogen interaction pathway, we found that ZmWRKY83 (Zm00001d038023), the homolog of AtWRKY33 in *Arabidopsis*, was always enriched with high protein abundance. Meanwhile, *ZmWRKY83* was continuously upregulated from 0 dpi to 2 dpi both in transcript and protein levels (Figure 6), which indicated that ZmWRKY83 was induced by *F. graminearum* infection and might play an important role in disease resistance. As a transcription factor, transcriptional activity is necessary. Therefore, we used the yeast two-hybrid system to verify the transcriptional activity of ZmWRKY83. The transformed yeast strain grew well on SD/-Leu/-Trp and SD/-Leu/-Trp/-His medium and appeared blue on the selective SD/-Leu/-Trp/-His/x-α-gal medium, which indicated that the ZmWRKY83 transcription factor had the transcriptional activation activity (Supplementary Figure 3).

**FIGURE 8:**
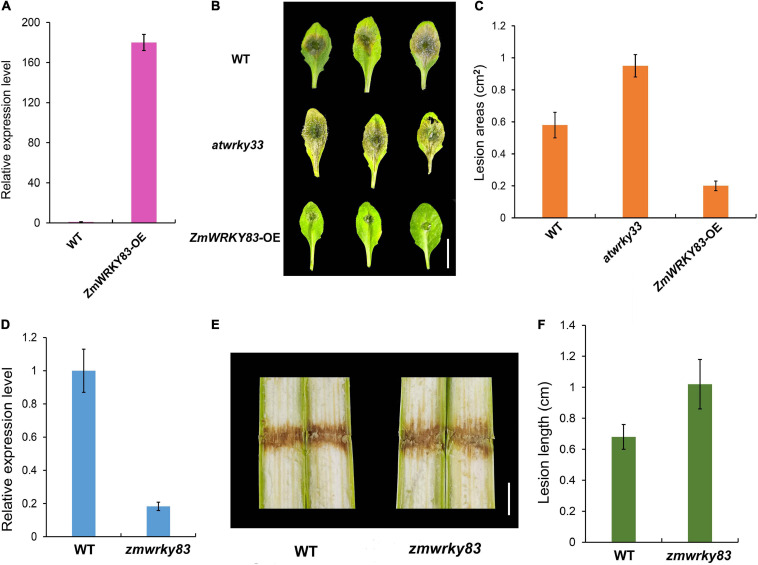
*ZmWRKY83* overexpression in transgenic *Arabidopsis* increases *B. cinerea* resistance. **(A)** The relative expression level of *ZmWRKY83* in Col-0 and *ZmWRKY83* overexpressing transgenic plants. **(B)** Altered disease resistance of Col-0, *atwrky33* mutant, and *ZmWRKY83* overexpressing transgenic plants against *B. cinerea*. Four-week-old plants were inoculated by *B. cinerea* spore suspension (10^6^ ml^–1^) and kept at high humidity. The experiments were repeated three times with similar results. **(C)** Measurement of lesion areas on *B. cinerea*-inoculated *Arabidopsis* leaves shown in panel **B**. The necrotic lesion areas on each leaf were measured using ImageJ. Data represent the mean ± SD. **(D)** The relative expression level of *ZmWRKY83* in B73 and *zmwrky83* mutant. **(E)** Altered disease resistance of B73, *zmwrky33* mutant against *F. graminearum*. V7 stage plants were inoculated by *F. graminearum* spore suspension (10^6^ ml^–1^) and kept at high humidity. **(F)** Measurement of lesion length on *F. graminearum*-inoculated maize stem shown in panel **E**. Data represent the mean ± SD.

To investigate the function of ZmWRKY83 in disease resistance, *ZmWRKY83* overexpressing transgenic plants were generated in *Arabidopsis* by *Agrobacterium*-mediated transformation (Figure 8A). Then, we conducted a *B. cinerea* infection assay in Col-0, *atwrky33* mutant, and *ZmWRKY83* overexpressing transgenic plants. Compared with Col-0 and *atwrky33* mutant, the resistibility of *ZmWRKY83*-OE plants to *B. cinerea* was relatively strong (Figure 8B and Supplementary Figure 4). In other words, the leaves of Col-0 and *atwrky33* mutants were more sensitive to *B. cinerea*, whereas overexpression of *ZmWRKY83* could enhance the resistance of *Arabidopsis* to this pathogen. Statistical analysis results showed that the size of the lesion area on Col-0 and *atwrky33* mutant leaves after *B. cinerea*-infected was more than the lesion area on *B. cinerea*-infected *ZmWRKY83*-OE leaves (Figure 8C). In addition, we got a Mutator insertion line of *ZmWRKY83*, in which *ZmWRKY83* showed low expression (Figure 8D). Then, we conducted *F. graminearum* infection assay in wild type B73 and *zmwrky83* mutant. The results showed that the *zmwrky83* mutant was more sensitive to *F. graminearum* infection (Figures 8E,F). These results suggested that ZmWRKY83 plays an important role in the plant defense system.

## Discussion

Plant diseases caused by pathogenic bacteria, such as fungi, bacteria, etc., have caused significant economic losses to important crops in the world (Ahuja et al., 2012). *Fusarium* species, such as *F. graminearum*, *Fusarium verticillioides*, and *Fusarium proliferatum*, are fungal pathogens associated with maize diseases (Dorn et al., 2011; Nguyen et al., 2016; Shu et al., 2017). *Fusarium* spp. can infect maize through all stages of growth and development and cause significant damage to different tissues of maize. For example, *F. graminearum* can cause ear rot and stem rot of maize and *F. graminearum* causes corn ear rot and stalk rot (Li et al., 2019). Previous studies focused more on-ear and kernel rot in maize, as mechanized harvesting has attracted much more attention, stalk rot has become more important.

In a previous study, global proteomics using iTRAQ technology were employed to study the early infection by *F. graminearum* in maize kernels. A subset of 878 proteins was identified between mock and treatment. Many proteins related to defense response after infection are induced, including chitinases, pathogenesis-related (PR) proteins, and some differentially expressed proteins (DEPs) between maize-resistant and sensitive-inbred lines (Mohammadi et al., 2011). This study aimed to identify the defense response proteins of maize to *F. graminearum* infection. Proteomics analysis of maize infected by *F. graminearum* in different time points was performed using the TMT technique (Figure 1). As a result, a total of 1,894 DEPs were identified, which included 1,177 upregulated and 717 downregulated proteins (Figure 2). Function analysis of the DEPs during *F. graminearum* infection showed that might modulate the immune system by slowing down plant growth and energy metabolism (Figures 3–5).

Phenylpropanoids are secondary metabolites of plants derived from aromatic amino acid phenylalanine in most plants or tyrosine in some monocotyledons (Deng and Lu, 2017). Phenylpropanoids can participate in local and systemic signal transduction induced by defense response genes (Dixon et al., 2002). In the process of protecting plants from pathogen infection, the key enzymes of the phenylalanine pathway, such as phenylalanine ammonia-lyase (PAL), peroxidase (POD), and polyphenol oxidase (PPO), are involved in the biosynthesis of lignin metabolites, which provides a structural barrier against fungi (Dixon et al., 2002; Mohammadi and Kazemi, 2002). In this study, we found that 29 upregulated proteins during *F. graminearum* infection were enriched in phenylpropanoid biosynthesis, including PODs, PALs, and other key enzymes (Supplementary Table 2).

In *Arabidopsis*, it is proved that calcium-dependent protein kinases (CDPKs) can mediate calcium-directed phosphorylation during plant defense activation and calmodulin-like (CML) proteins are important mediators of Ca^2+^-dependent signals during the immune response to plant pathogens (Harmon et al., 2000; Harper and Harmon, 2005; Leba et al., 2012). In plant-pathogen interaction pathway from upregulated DEPs enrichment analysis, two CMLs (Zm00001d029028, Zm00001d010649) and three CDPKs (Zm00001d021835, Zm00001d009708, and Zm00001d013030) were specifically upregulated during *F. graminearum* infection, which emphasizes the possible role of Ca^2+^-dependent signaling in the resistance to *F. graminearum* infection.

Production of PR proteins is a defense strategy often adopted by plants to resist the invasion of pathogens (Sels et al., 2008). PR protein has direct antibacterial properties, and some PR proteins can also inhibit pathogens by regulating key genes in the host defense system (van Loon et al., 2006). ZmPR10 plays a role in maize host resistance to *A. flavus* infection and aflatoxin contamination (Chen et al., 2006). The PR maize seed (PRms, *Zm00001d009296*) gene is proven to be involved in resistance to *A. flavus* and *F. verticillioides* infection (Shu et al., 2015; Majumdar et al., 2017a). In this study, ZmPR10, ZmPRms, and several PR proteins were upregulated both in transcript and protein levels during *F. graminearum* infection (Figure 6 and Supplementary Table 2), which suggested that ZmPRms played a key role in maize ear and stalk rot diseases and was a broad-spectrum disease resistance gene.

Terpenoids are the most diverse in structure, and they act as plant antitoxins in direct defense of plants or signal in indirect defense reactions (Cheng et al., 2007). Terpenoids, monoterpenes, sesquiterpenes, semiterpenoids, and diterpenoids can participate in the protection of plants against abiotic and biotic stresses (Loreto et al., 2014). In this study, 13 proteins were detected and enriched in terpenoids and diterpenoids biosynthesis during *F. graminearum’s* later infection (Figure 6 and Supplementary Table 2). These results indicated that the proteins involved in terpenoids and diterpenoids biosynthesis might be important to the resistance of fungal infection. Xylanase inhibitor proteins (XIP) are reported to be involved in the plant defense mechanisms against fungal pathogens and has been fully characterized in several model plant species (Misas-Villamil and van der Hoorn, 2008; Vasconcelos et al., 2011; Sun et al., 2018). Two XIPs were detected during *F. graminearum* infection and upregulated (Supplementary Table 2), which suggested that these XIPs may act as a barrier to prevent fungal pathogens secreting xylanase from degrading cell walls.

Several studies confirm that WRKY transcription factors are key regulators in response to abiotic stresses (Rushton et al., 2010). In maize, ZmWRKY17 is involved in regulating the expression of some ABA and stress response genes and negatively reduces ABA sensitivity, and regulates salt tolerance (Cai et al., 2017). ZmWRKY106 participates in a variety of abiotic stress response pathways and plays an active role under drought and high-temperature stress (Wang et al., 2018a). ZmWRKY33 can be induced by salt stress, drought stress, cold treatment, and ABA treatment, and overexpression in *Arabidopsis* plants can enhance salt tolerance (Li et al., 2013). ZmWRKY4 can be induced by Cd, and its role in regulating the expression and activity of SOD and APX in maize was studied by transient expression analysis based on maize protoplasts (Hong et al., 2017). However, the role of WRKY transcription factors in biotic stress is few studied in maize.

AtWRKY33 is involved in regulating gene transcription activity and it plays an important role in plant defense against *B. cinerea* (Birkenbihl et al., 2012, 2017a; Sham et al., 2017). In this study, we found a WRKY transcription factor ZmWRKY83 (homolog of AtWRKY33) that may play a key role in maize resistance to *F. graminearum* infection by proteomics. A recent study shows that *ZmWRKY52*, *ZmWRKY71*, and *ZmWRKY83* genes display significantly differential expression levels among the QTL-NILs, which indicated that these maize WRKY transcription factors may be involved in maize resistance to *A. flavus* (Parish et al., 2019). However, there is no direct experimental evidence that ZmWRKY83 was involved in resistance to pathogen infection. In this study, ZmWRKY83 protein was first identified to be associated with *F. graminearum* infection (Figure 6). Through overexpression of *ZmWRKY83* in Arabidopsis and *B. cinerea* infection assay, it is shown that this gene was closely related to plant disease resistance (Figure 8), which indicated that *ZmWRKY83* could be applied to molecular genetic breeding of maize disease-resistant varieties.

In summary, we used quantitative proteomics technology to compare the differential protein abundance after *F. graminearum* infection. Through a TMT-based proteomic approach, we identified 1,894 DEPs from maize stems challenged with the necrotrophic fungus pathogen *F. graminearum*. GO and KEGG pathway enrichment analysis showed that proteins related to plant-pathogen interaction and secondary metabolites biosynthesis were upregulated, whereas energy metabolism and plant development were downregulated to resist *F. graminearum* infection. In addition, we identified a disease-resistant transcription factor and proved that ZmWRKY83 plays an important role in plant immunity against fungal infection. Collectively, our results would also provide compelling evidence that the plant immune-related proteins are involved in the response to *F. graminearum* infection, which complements the latest knowledge about the maize resistance to *F. graminearum* infestation.

## Materials and Methods

### Plant Materials and Treatments

The maize inbred line B73 was grown in the greenhouse in Baoding (Hebei Agricultural University, Hebei province, China). Maize *zmwrky83* mutant was obtained from the ChinaMu project (insertion site: chr 6, 145531076^1^) (Liang et al., 2019). *F. gramincarum* PH-1 was grown on PDA plates at 28°C for 6 days. Conidial suspensions were harvested by adding sterile distilled water containing 0.05% (v/v) Triton X-100 and scraping the plates using a glass spreader. The concentration of conidia was quantified using a hemocytometer and diluted to 1 × 10^6^ spores/ml for inoculation. Maize stem was inoculated at the second or third internode using a sterile micropipet tip (10 mm hole depth) above the soil line, followed by injection of 20 μl macroconidia suspension. The plants were kept growing with the wounds being covered by sterile gauze to maintain moisture and avoid contamination from other organisms.

### Protein Extraction, Quantification, and Digestion

The sample was ground into powder by liquid nitrogen and then transferred to a 5 ml centrifuge tube. Then, lysis buffer (including 1% TritonX-100, 10 mM dithiothreitol, and 1% protease inhibitor cocktail, 50 μM PR-619,3 μM TSA,50 mM NAM, and 2 mM EDTA) was added to the powder, followed by sonication three times on ice using a high-intensity ultrasonic processor (Scientz). After an equal volume of Tris-saturated phenol (pH 8.0) was added, the mixture was further vortexed for 5 min. After centrifugation (4°C, 10 min, 5,000 *g*), the upper phenol phase was transferred to a new centrifuge tube. Proteins were precipitated by adding at least four volumes of ammonium sulfate-saturated methanol and incubated at −20°C for at least 6 h. After centrifugation at 4°C for 10 min, the supernatant was discarded. The remaining precipitate was washed with ice-cold methanol once, followed by ice-cold acetone three times. The protein was resuspended in 8 M urea (Sigma), and then the redissolved protein concentration was detected with a BCA kit (Beyotime) according to the instructions of the manufacturer.

The protein solution was reduced with 5 mM dithiothreitol (Sigma) for 30 min at 56°C and alkylated with 11 mM iodoacetamide (Sigma) for 15 min in darkness at room temperature. The protein sample was then diluted by adding 100 mM TEAB to urea concentration less than 2 M. Trypsin (Promega) was added at 1:50 trypsin-to-protein mass ratio for the first digestion overnight and 1:100 trypsin-to-protein mass ratio for a second 4-h-digestion.

### TMT Labeling and Peptide Fractionation

The peptide was desalted by Strata X C18 SPE column (Phenomenex) and vacuum-dried after trypsin digestion. According to the manufacturer’s protocol, peptide was reconstituted in 0.5 M TEAB and processed with TMT kit (Thermo Fisher Scientific^2^). Briefly, 1 unit of TMT reagent was thawed and reconstituted in acetonitrile. Then, the peptide mixture was incubated at room temperature for 2 h, and the pool was centrifuged in a vacuum, desalted, and dried. The tryptic peptides were fractionated into fractions by high pH reverse-phase HPLC using Agilent 300 Extend C18 column (5 μm particles, 4.6 mm ID, and 250 mm length). Then, the peptides were combined into 18 components with a gradient of 8–32% acetonitrile (pH 9.0) and dried by vacuum centrifugation.

### LC-MS-MS Analysis

Trypsin peptides are dissolved in 0.1% formic acid and directly loaded on a self-made reversed-phase analytical column (15 cm length, 75 μm i.d.). The gradient of solvent B (0.1% formic acid in 98% acetonitrile) was gradually increased from 6% to 23% through 26 min, increased from 23% to 35% over 8 min, climbing to 80% in 3 min, and then maintained at 80% for 3 min, all at a constant flow rate of 400 nl/min on an EASY-nLC 1000 UPLC system. The separated peptides were subjected to NSI source followed by tandem mass spectrometry (MS/MS) in Q Exactive^TM^ Plus (Thermo) coupled online to the UPLC. A 2.0 kV electrospray voltage was applied. At 70,000 resolutions, the intact peptides were detected in the Orbitrap, with 350–1800 *m/z* full scan range. Up to 20 most abundant precursors were then selected for further MS/MS analyses with 30-s dynamic exclusion. The HCD fragmentation was performed at a normalized collision energy of 28%. The fragments were detected in the Orbitrap at a resolution of 17,500. The fixed first mass was set as 100 *m/z*. The automatic gain control target was set at 5E4, with an intensity threshold of 1E4 and a maximum injection time of 200 ms.

### Database Search and Bioinformatics Analysis

Tandem mass spectrometry data were processed by the Maxquant search engine (version 1.5.2.8). MS/MS were searched against MaizeGDB (version 4, 131,585 sequences) database concatenated with reverse decoy database. Trypsin/P was designated as lyase, allowing up to two missing cleavages. The mass tolerance of precursor ions is set to 20 ppm in the first search, 5 ppm in the main search, and 0.02 Da in the fragment ions. The aminoformyl methyl on Cys was designated as a fixed modification, and the oxidation on Met was designated as a variable modification. The minimum score for peptides was set > 40 and FDR was adjusted to < 1%.

Functional annotations of DEPs were performed using agriGOv2 (Tian et al., 2017). The KEGG was used to predict the metabolic pathways and biochemical signals transduction pathways (Kanehisa and Goto, 2000). A *p*-value < 0.05 (Fisher’s exact test) was used as the threshold to determine the significant enrichment of GO and KEGG pathways.

### RNA Extraction and Quantitative Real-Time PCR

The same samples used in proteomics were homogenized in liquid nitrogen before RNA isolation. Total RNA of the samples was isolated using TRIZOL (Invitrogen) and purified using Qiagen RNeasy columns. qRT-PCR was conducted using actin as an internal reference and cDNAs from samples collected at different time points as the template with TransStart Tip Green qPCR SuperMix according to the manufacturer’s instructions. Furthermore, comparative Ct analysis (2-^ΔΔ^Ct) of each gene in B73 and its relative expression levels at different time points were employed, and quantitative data were expressed as mean ± SEM. Primers used in this study were designed with Primer 6.0 software and listed in Supplementary Table 3.

### Detection of ZmWRKY83 Transcription Activation Activity

To detect the transcriptional activation of ZmWRKY83, the CDS of ZmWRKY83 were amplified and cloned into the yeast vector pGBKT7 (Clontech) to obtain BD- ZmWRKY83 construct. BD- ZmWRKY83 construct and empty vector pGBKT7 were, respectively, transformed into yeast strain AH109. A transformed single colony (2 mm diameter) grown on selection medium for 3 days was resuspended in 100 μl of autoclaved distilled H_2_O, and 10 μl of resuspended cells was plated on SD/-Leu-Trp, SD/-Leu-Trp-His/x-α-gal for 3–5 days.

### Construction of ZmWRKY83 Overexpression Plants in *Arabidopsis*

A full-length ZmWRKY83 CDS sequence was amplified by PCR with a specific primer (Supplementary Table 3). After sequence verification, the fragment was inserted into the binary vector pCAMBIA-1300. The recombinant plasmid was introduced into Columbia-0 (Col-0) plants by *Agrobacterium* using floral dipping. The transformed seeds were selected on a half-strength MS medium containing Basta.

### *B. cinerea* Inoculation

*Botrytis cinerea* strain B05.10 was cultured on PDA (potato dextrose agar) media for 5–10 days at 18–22°C. Conidia inoculums of *B. cinerea* were prepared by collecting conidia in sterile water, filtered to remove mycelia, and suspended to a final concentration of 10^6^ conidia/ml. For resistance analysis, the single leaf of plants was inoculated with 5 μl conidia suspension of *B. cinerea*. Inoculated plants were kept in a growth chamber with high humidity for 24 h and then transferred to normal conditions. Lesion formation was monitored at 7 days postinoculation (dpi). Trypan blue staining was performed as described by the previous study (Hao et al., 2013).

## Data Availability Statement

The datasets presented in this study can be found in online repositories. The names of the repository/repositories and accession number(s) can be found below: The datasets generated and analyzed during the current study are available in the ProteomeXchange Consortium via the PRIDE partner repository with the dataset identifier PXD024342.

## Author Contributions

KZ, JX, and JD designed the experiments, analyzed the data, and wrote the study. HB, HS, and JZ analyzed the data, performed phenotype analysis, and qRT-PCR assays. XP, HC, and LY performed transcription activation activity assays. HB and HC constructed the ZmWRKY83 overexpression plants in *Arabidopsis*. KZ, HB, and XP identified the WRKY33 mutants and performed *B. cinerea* inoculation. All authors read and approved the contents of this study.

## Conflict of Interest

The authors declare that the research was conducted in the absence of any commercial or financial relationships that could be construed as a potential conflict of interest.

## Publisher’s Note

All claims expressed in this article are solely those of the authors and do not necessarily represent those of their affiliated organizations, or those of the publisher, the editors and the reviewers. Any product that may be evaluated in this article, or claim that may be made by its manufacturer, is not guaranteed or endorsed by the publisher.

## Footnotes

1
http://chinamu.jaas.ac.cn/Default.html

2
https://www.thermofisher.com/

## Supplementary Material

Supplementary Figure 1 Principal component analysis (PCA) of the total proteome data from maize stem at 0, 1, and 2 days after *F. graminearum* infection. Protein samples at each time point have three replicates.

Supplementary Figure 2 Venn diagram analysis of the DEPs that were up- or down-regulated in maize stem at 0, 1, and 2 days post *F. graminearum* infection. **(A)** Venn diagram of up-regulated proteins. **(B)** Venn diagram of down-regulated proteins.

Supplementary Figure 3 Transactivation activity analysis of ZmWRKY83 in yeast. pGBKT7 empty vector (BD) was used as the negative control. The results were determined by the growth of the yeast strains SD/-Leu/-Trp, SD/-Leu/-Trp/-His and SD/-Trp/-His/-Ade/x-α-gal media for 3–5 days at 30°C.

Supplementary Figure 4 Trypan blue staining of leaves from Col-0, *atwrky33* mutant, and *ZmWRKY83* overexpressing transgenic plants at 7 days after *B. cinerea* infection showing the extent of death cell triggered by pathogen infection.

Supplementary Table 1 MS/MS spectrum database search analysis of proteins identified during *F. graminearum* infection at 0, 1, and 2 dpi in maize stem.

Supplementary Table 2 The list of DEPs induced by *F. graminearum* infection at 0, 1, and 2 dpi in maize stem.

Supplementary Table 3 Primers sequences used for qRT-PCR amplification of selected genes for validation.

Supplementary Table 4 Quantitative real-time PCR (qRT-PCR) results of the proteins enriched in plant-pathogen interaction pathway, terpenoid backbone, and diterpenoid biosynthesis pathway in B73 and Mo17. Data represent the mean ± SD.

Supplementary Table 5 Quantitative real-time PCR results of the proteins enriched in plant-pathogen interaction pathway, terpenoid backbone, and diterpenoid biosynthesis pathway in 0, 1, 2, and 3 dpi in maize stem with *F. graminearum* infection. Data represent the mean ± SD.

## References

[B1] Ahuja I. Kissen R. Bones A. M. (2012). Phytoalexins in defense against pathogens. *Trends Plant Sci.* 17 73–90. 10.1016/j.tplants.2011.11.002 22209038

[B2] Akira S. Uematsu S. Takeuchi O. (2006). Pathogen recognition and innate immunity. *Cell* 124 783–801. 10.1016/j.cell.2006.02.015 16497588

[B3] Birkenbihl R. P. Diezel C. Somssich I. E. (2012). *Arabidopsis* WRKY33 is a key transcriptional regulator of hormonal and metabolic responses toward *Botrytis cinerea* infection. *Plant Physiol.* 159 266–285. 10.1104/pp.111.192641 22392279 PMC3375964

[B4] Birkenbihl R. P. Kracher B. Roccaro M. Somssich I. E. (2017a). Induced genome-wide binding of three Arabidopsis WRKY transcription factors during early MAMP-triggered immunity. *Plant Cell* 29 1175–1175. 10.1105/tpc.17.00278 28011690 PMC5304350

[B5] Birkenbihl R. P. Kracher B. Somssich I. E. (2017b). Induced genome-wide binding of three *Arabidopsis* WRKY transcription factors during early MAMP-triggered immunity. *Plant Cell* 29 20–38. 10.1105/tpc.16.00681 28011690 PMC5304350

[B6] Cai R. H. Dai W. Zhang C. S. Wang Y. Wu M. Zhao Y. (2017). The maize WRKY transcription factor ZmWRKY17 negatively regulates salt stress tolerance in transgenic *Arabidopsis* plants. *Planta* 246 1215–1231. 10.1007/s00425-017-2766-9 28861611

[B7] Chen Q. Song J. Du W. P. Xu L. Y. Jiang Y. Zhang J. (2017). Identification, Mapping, and Molecular Marker Development for Rgsr8.1: A new quantitative trait locus conferring resistance to *Gibberella* stalk rot in maize (*Zea mays* L.). *Front. Plant Sci.* 8:1355. 10.3389/fpls.2017.01355 28824686 PMC5540892

[B8] Chen Z. Y. Brown R. L. Rajasekaran K. Damann K. E. Cleveland T. E. (2006). Identification of a maize kernel pathogenesis-related protein and evidence for its involvement in resistance to *Aspergillus flavus* infection and aflatoxin production. *Phytopathology* 96 87–95. 10.1094/PHYTO-96-0087 18944208

[B9] Cheng A. X. Lou Y. G. Mao Y. B. Lu S. Wang L. J. Chen X.-Y. (2007). Plant terpenoids: biosynthesis and ecological functions. *J. Integr. Plant Biol.* 49 179–186. 10.1111/j.1744-7909.2007.00395.x

[B10] Cui H. Tsuda K. Parker J. E. (2015). Effector-triggered immunity: from pathogen perception to robust defense. *Annu. Rev. Plant Biol.* 66 487–511. 10.1146/annurev-arplant-050213-040012 25494461

[B11] Deng Y. Lu S. (2017). Biosynthesis and regulation of phenylpropanoids in plants. *Crit. Rev. Plant Sci.* 36 257–290. 10.1080/07352689.2017.1402852

[B12] Dixon R. A. Achnine L. Kota P. Liu C. J. Reddy M. S. S. Wang L. J. (2002). The phenylpropanoid pathway and plant defence - a genomics perspective. *Mole. Plant Pathol.* 3 371–390. 10.1046/j.1364-3703.2002.00131.x 20569344

[B13] Dorn B. Forrer H. R. Jenny E. Wettstein F. E. Bucheli T. D. Vogelgsang S. (2011). *Fusarium* species complex and mycotoxins in grain maize from maize hybrid trials and from grower’s fields. *J. Appl. Microbiol.* 111 693–706. 10.1111/j.1365-2672.2011.05091.x 21714835

[B14] Dunkley T. P. J. Hester S. Shadforth I. P. Runions J. Weimar T. Hanton S. L. (2006). Mapping the *Arabidopsis* organelle proteome. *Proc. Natl. Acad. Sci. U S A* 103 6518–6523. 10.1073/pnas.0506958103 16618929 PMC1458916

[B15] Gai X. T. Dong H. Y. Wang S. N. Liu B. Zhang Z. R. Li X. Y. (2018). Infection cycle of maize stalk rot and ear rot caused by *Fusarium verticillioides*. *PLo*S *One* 13:7. 10.1371/journal.pone.0201588 30063754 PMC6067754

[B16] Han X. F. Li S. Zhang M. Yang L. Y. Liu Y. D. Xu J. (2019). Regulation of GDSL lipase gene expression by the MPK3/MPK6 cascade and its downstream WRKY transcription factors in *Arabidopsis* Immunity. *Mole. Plant-Microb. Interac.* 32 673–684. 10.1094/Mpmi-06-18-0171-R 30598046

[B17] Hao C. C. Jia J. Chen Z. Xing J. H. Weng Q. Y. Wang F. R. (2013). Functional analysis of BT4 of *Arabidopsis thaliana* in resistance against *Botrytis cinerea*. *Austr. Plant Pathol.* 42 393–401. 10.1007/s13313-013-0202-6

[B18] Harmon A. C. Gribskov M. Harper J. F. (2000). CDPKs - a kinase for every Ca2+ signal? *Trends Plant Sci.* 5 154–159. 10.1016/s1360-1385(00)01577-6 10740296

[B19] Harper J. F. Harmon A. (2005). Plants, symbiosis and parasites: A calcium signalling connection. *Nat. Rev. Mole. Cell Biol.* 6 555–566. 10.1038/nrm1679 16072038

[B20] Hong C. Y. Cheng D. Zhang G. Q. Zhu D. D. Chen Y. H. Tan M. P. (2017). The role of ZmWRKY4 in regulating maize antioxidant defense under cadmium stress. *Biochemical. Biophys. Comm.* 482 1504–1510. 10.1016/j.bbrc.2016.12.064 27956180

[B21] Hu X. L. Li N. Wu L. J. Li C. Q. Li C. H. Zhang L. (2015). Quantitative iTRAQ-based proteomic analysis of phosphoproteins and ABA-regulated phosphoproteins in maize leaves under osmotic stress. *Sci. Rep.* 5:15626. 10.1038/Srep15626 26503333 PMC4650667

[B22] Huang X. L. Liu L. X. Zhai Y. H. Liu T. Chen J. (2009). Proteomic comparison of four maize inbred lines with different levels of resistance to Curvularia lunata (Wakker) Boed infection. *Prog. Nat. Sci. Mater. Internat.* 19 353–358. 10.1016/j.pnsc.2008.04.020

[B23] Kanehisa M. Goto S. (2000). KEGG: kyoto encyclopedia of genes and genomes. *Nucleic Acids Res.* 28 27–30. 10.1093/nar/28.1.27 10592173 PMC102409

[B24] Kazan K. Lyons R. (2014). Intervention of Phytohormone Pathways by Pathogen Effectors. *Plant Cell* 26 2285–2309. 10.1105/tpc.114.125419 24920334 PMC4114936

[B25] Lade S. B. Roman C. Cueto-Ginzo A. I. Serrano L. Sin E. Achon M. A. (2018). Host-specific proteomic and growth analysis of maize and tomato seedlings inoculated with Azospirillum brasilense Sp7. *Plant Physiol. Biochem.* 129 381–393. 10.1016/j.plaphy.2018.06.024 29945074

[B26] Leba L. J. Cheval C. Ortiz-Martin I. Ranty B. Beuzon C. R. Galaud J. P. (2012). CML9, an Arabidopsis calmodulin-like protein, contributes to plant innate immunity through a flagellin-dependent signalling pathway. *The Plant Journal* 71 976–989. 10.1111/j.1365-313X.2012.05045.x 22563930

[B27] Li H. Gao Y. Xu H. Dai Y. Deng D. Q. Chen J. M. (2013). ZmWRKY33, a WRKY maize transcription factor conferring enhanced salt stress tolerances in Arabidopsis. *Plant Growth Regul.* 70 207–216. 10.1007/s10725-013-9792-9

[B28] Li L. N. Qu Q. Cao Z. Y. Guo Z. Y. Jia H. Liu N. (2019). The relationship analysis on corn stalk rot and ear rot according to *Fusarium* species and Fumonisin contamination in kernels. *Toxins* 11 6. 10.3390/toxins11060320 31195636 PMC6628441

[B29] Liang L. Zhou L. Tang Y. Li N. Song T. Shao W. (2019). A sequence-indexed Mutator insertional library for maize functional genomics study. *Plant Physiol.* 181 1404–1414. 10.1104/pp.19.00894 31636104 PMC6878021

[B30] Liu S. A. Ziegler J. Zeier J. Birkenbihl R. P. Somssich I. E. (2017). Botrytis cinerea B05.10 promotes disease development in *Arabidopsis* by suppressing WRKY33-mediated host immunity. *Plant Cell and Environment* 40 2189–2206. 10.1111/pce.13022 28708934

[B31] Llorca C. M. Potschin M. Zentgraf U. (2014). bZIPs and WRKYs: two large transcription factor families executing two different functional strategies. *Front. Plant Sci.* 5:169. 10.3389/fpls.2014.00169 24817872 PMC4012195

[B32] Loreto F. Dicke M. Schnitzler J. P. Turlings T. C. (2014). Plant volatiles and the environment. *Plant Cell and Environment* 37 1905–1908. 10.1111/pce.12369 24811745

[B33] Ma C. Ma X. Yao L. Liu Y. Du F. Yang X. (2017). qRfg3, a novel quantitative resistance locus against *Gibberella* stalk rot in maize. *Theoret. Appl. Genet.* 130 1723–1734. 10.1007/s00122-017-2921-5 28555262

[B34] Majumdar R. Rajasekaran K. Sickler C. Lebar M. Musungu B. M. Fakhoury A. M. (2017a). The pathogenesis-related maize seed (PRms) gene plays a role in resistance to *Aspergillus flavus* infection and aflatoxin contamination. *Front. Plant Sci.* 8:1758. 10.3389/fpls.2017.01758 29089952 PMC5651032

[B35] Majumdar R. Rajasekaran K. Sickler C. Lebar M. Musungu B. M. Fakhoury A. M. (2017b). The pathogenesis-related maize seed (PRms) gene plays a role in resistance to *Aspergillus flavus* infection and aflatoxin contamination. *Front. Plant Sci.* 8:1758. 10.3389/Fpls.2017.01758 29089952 PMC5651032

[B36] Merz P. R. Moser T. Holl J. Kortekamp A. Buchholz G. Zyprian E. (2015). The transcription factor VvWRKY33 is involved in the regulation of grapevine (Vitis vinifera) defense against the oomycete pathogen Plasmopara viticola. *Physiol. Plant.* 153 365–380. 10.1111/ppl.12251 25132131

[B37] Mesterhazy A. Lemmens M. Reid L. M. (2012). Breeding for resistance to ear rots caused by Fusarium spp. in maize - a review. *Plant Breed.* 131 1–19. 10.1111/j.1439-0523.2011.01936.x

[B38] Misas-Villamil J. C. van der Hoorn R. A. L. (2008). Enzyme–inhibitor interactions at the plant–pathogen interface. *Curr. Opin. Plant Biol.* 11 380–388. 10.1016/j.pbi.2008.04.007 18550418

[B39] Mohammadi M. Anoop V. Gleddie S. Harris L. J. (2011). Proteomic profiling of two maize inbreds during early gibberella ear rot infection. *Proteomics* 11 3675–3684. 10.1002/pmic.201100177 21751381

[B40] Mohammadi M. Kazemi H. (2002). Changes in peroxidase and polyphenol oxidase activities in susceptible and resistant wheat heads inoculated with *Fusarium graminearum* and induced resistance. *Plant Sci.* 162 491–498. 10.1016/S0168-9452(01)00538-6

[B41] Nguyen T. T. X. Dehne H. W. Steiner U. (2016). Histopathological assessment of the infection of maize leaves by *Fusarium graminearum*, *F. proliferatum*, and *F. verticillioides*. *Fungal Biol.* 120 1094–1104. 10.1016/j.funbio.2016.05.013 27567716

[B42] Parish F. Williams W. P. Windham G. L. Shan X. Y. (2019). Differential expression of signaling pathway genes associated with aflatoxin reduction quantitative trait loci in maize (*Zea mays* L.). *Front. Microb.* 10:2683. 10.3389/Fmicb.2019.02683 31849861 PMC6901933

[B43] Pe M. E. Gianfranceschi L. Taramino G. Tarchini R. Angelini P. Dani M. (1993). Mapping quantitative trait loci (QTLs) for resistance to Gibberella zeae infection in maize. *Mole. Gen. Genet.* 241 11–16. 10.1007/bf00280195 7901750

[B44] Pechanova O. Pechan T. Williams W. P. Luthe D. S. (2011). Proteomic analysis of the maize rachis: Potential roles of constitutive and induced proteins in resistance to Aspergillus flavus infection and aflatoxin accumulation. *Proteomics* 11 114–127. 10.1002/pmic.201000368 21182199

[B45] Pechanova O. Takac T. Samaj J. Pechan T. (2013). Maize proteomics: An insight into the biology of an important cereal crop. *Proteomics* 13 637–662. 10.1002/pmic.201200275 23197376

[B46] Rushton P. J. Somssich I. E. Ringler P. Shen Q. X. J. (2010). WRKY transcription factors. *Trends Plant Sci.* 15 247–258. 10.1016/j.tplants.2010.02.006 20304701

[B47] Santiago R. Reid L. M. Arnason J. T. Zhu X. Martinez N. Malvar R. A. (2007). Phenolics in maize genotypes differing in susceptibility to *Gibberella* stalk rot (*Fusarium graminearum* Schwabe). *J. Agricult. Food Chem.* 55 5186–5193. 10.1021/jf070641e 17547419

[B48] Sels J. Mathys J. De Coninck B. M. Cammue B. P. De Bolle M. F. (2008). Plant pathogenesis-related (PR) proteins: a focus on PR peptides. *Plant Physiol. Biochem.* 46 941–950. 10.1016/j.plaphy.2008.06.011 18674922

[B49] Sham A. Moustafa K. Al-Shamisi S. Alyan S. Iratni R. AbuQamar S. (2017). Microarray analysis of Arabidopsis WRKY33 mutants in response to the necrotrophic fungus *Botrytis cinerea*. *PLoS One* 12:e0172343. 10.1371/journal.pone.0172343 28207847 PMC5313235

[B50] Shu X. Livingston D. P. III Franks R. G. Boston R. S. Woloshuk C. P. Payne G. A. (2015). Tissue-specific gene expression in maize seeds during colonization by *Aspergillus flavus* and *Fusarium verticillioides*. *Mole. Plant Pathol.* 16 662–674. 10.1111/mpp.12224 25469958 PMC6638326

[B51] Shu X. M. Livingston D. P. Woloshuk C. P. Payne G. A. (2017). Comparative histological and transcriptional analysis of maize kernels infected with *Aspergillus flavus* and *Fusarium verticillioides*. *Front. Plant Sci.* 8:2075. 10.3389/Fpls.2017.02075 29270183 PMC5723656

[B52] Sun R. J. Xu Y. Hou C. X. Zhan Y. H. Liu M. Q. Weng X. Y. (2018). Expression and characteristics of rice xylanase inhibitor OsXIP, a member of a new class of antifungal proteins. *Biologia Plantar.* 62 569–578. 10.1007/s10535-018-0787-2

[B53] Thomma B. P. Nurnberger T. Joosten M. H. (2011). PAMPs and effectors: the blurred PTI-ETI dichotomy. *Plant Cell* 23 4–15. 10.1105/tpc.110.082602 21278123 PMC3051239

[B54] Tian T. Liu Y. Yan H. You Q. Yi X. Du Z. (2017). AgriGO v2.0: a GO analysis toolkit for the agricultural community, 2017 update. *Nucleic Acids Res.* 45 W122–W129. 10.1093/nar/gkx382 28472432 PMC5793732

[B55] Tsuda K. Katagiri F. (2010). Comparing signaling mechanisms engaged in pattern-triggered and effector-triggered immunity. *Curr. Opin. Plant Biol.* 13 459–465. 10.1016/j.pbi.2010.04.006 20471306

[B56] van Loon L. C. Rep M. Pieterse C. M. (2006). Significance of inducible defense-related proteins in infected plants. *Annu. Rev. Phytopathol.* 44 135–162. 10.1146/annurev.phyto.44.070505.143425 16602946

[B57] Vasconcelos E. A. Santana C. G. Godoy C. V. Seixas C. D. Silva M. S. Moreira L. R. (2011). A new chitinase-like xylanase inhibitor protein (XIP) from coffee (*Coffea arabica*) affects Soybean Asian rust (*Phakopsora pachyrhizi*) spore germination. *BMC Biotechnol.* 11:14. 10.1186/1472-6750-11-14 21299880 PMC3045311

[B58] Walley J. W. Shen Z. X. McReynolds M. R. Schmelz E. A. Briggs S. P. (2018). Fungal-induced protein hyperacetylation in maize identified by acetylome profiling. *Proc. Natl. Acad. Sci. U S A* 115 210–215. 10.1073/pnas.1717519115 29259121 PMC5776827

[B59] Wang C. T. Ru J. N. Liu Y. W. Li M. Zhao D. Yang J. F. (2018a). Maize WRKY Transcription Factor ZmWRKY106 Confers Drought and Heat Tolerance in Transgenic Plants. *Internat. J. Mole. Sci.* 19 3046. 10.3390/Ijms19103046 30301220 PMC6213049

[B60] Wang S. X. Chen Z. Tian L. Ding Y. Z. Zhang J. Zhou J. L. (2019). Comparative proteomics combined with analyses of transgenic plants reveal ZmREM1.3 mediates maize resistance to southern corn rust. *Plant Biotechnol. J.* 17 2153–2168. 10.1111/pbi.13129 30972847 PMC6790363

[B61] Wang Y. M. Schuck S. Wu J. N. Yang P. Doring A. C. Zeier J. (2018b). A MPK3/6-WRKY33-ALD1-pipecolic acid regulatory loop contributes to systemic acquired resistance. *Plant Cell* 30 2480–2494. 10.1105/tpc.18.00547 30228125 PMC6241261

[B62] Wang Z. Fang H. D. Chen Y. Chen K. P. Li G. Y. Gu S. L. (2014). Overexpression of BnWRKY33 in oilseed rape enhances resistance to *Sclerotinia sclerotiorum*. *Mole. Plant Pathol.* 15 677–689. 10.1111/mpp.12123 24521393 PMC6638750

[B63] Yang Q. Yin G. Guo Y. Zhang D. Chen S. Xu M. (2010). A major QTL for resistance to *Gibberella* stalk rot in maize. *Theoret. Appl. Genet.* 121 673–687. 10.1007/s00122-010-1339-0 20401458

[B64] Ye J.-R. Zhong T. Zhang D. Ma C. Wang L. Yao L. (2018). The auxin-regulated protein ZmAuxRP1 coordinates the balance between root growth and stalk-rot disease resistance in maize. *Molecul. Plant* 2018:005. 10.1016/j.molp.2018.10.005 30853061

[B65] Yue R. Q. Lu C. X. Han X. H. Guo S. L. Yan S. F. Liu L. (2018). Comparative proteomic analysis of maize (*Zea mays* L.) seedlings under rice black-streaked dwarf virus infection. *BMC Plant Biol* 18:191. 10.1186/S12870-018-1419-X 30208842 PMC6136180

[B66] Zhang D. Liu Y. Guo Y. Yang Q. Ye J. Chen S. (2012). Fine-mapping of qRfg2, a QTL for resistance to *Gibberella* stalk rot in maize. *Theoret. Appl. Genet.* 124 585–596. 10.1007/s00122-011-1731-4 22048640

[B67] Zhang Y. J. Wei M. Y. Liu A. L. Zhou R. Li D. H. Dossa K. (2019). Comparative proteomic analysis of two sesame genotypes with contrasting salinity tolerance in response to salt stress. *J. Proteom.* 201 73–83. 10.1016/j.jprot.2019.04.017 31009803

[B68] Zheng Z. Y. Abu Qamar S. Chen Z. X. Mengiste T. (2006). Arabidopsis WRKY33 transcription factor is required for resistance to necrotrophic fungal pathogens. *Plant J.* 48 592–605. 10.1111/j.1365-313X.2006.02901.x 17059405

[B69] Zhou J. Wang J. Zheng Z. Y. Fan B. F. Yu J. Q. Chen Z. X. (2015). Characterization of the promoter and extended C-terminal domain of *Arabidopsis* WRKY33 and functional analysis of tomato WRKY33 homologues in plant stress responses. *J. Exp. Bot.* 66 4567–4583. 10.1093/jxb/erv221 25969555 PMC4507763

