# Supplementary figures and images for: Comparative Proteomic Analysis of the Defense Response to Gibberella Stalk Rot in Maize and Reveals That ZmWRKY83 Is Involved in Plant Disease Resistance
Source: Front Plant Sci. 2021 Aug 13;12:694973. doi: 10.3389/fpls.2021.694973 (PMC8417113; doi:10.3389/fpls.2021.694973)

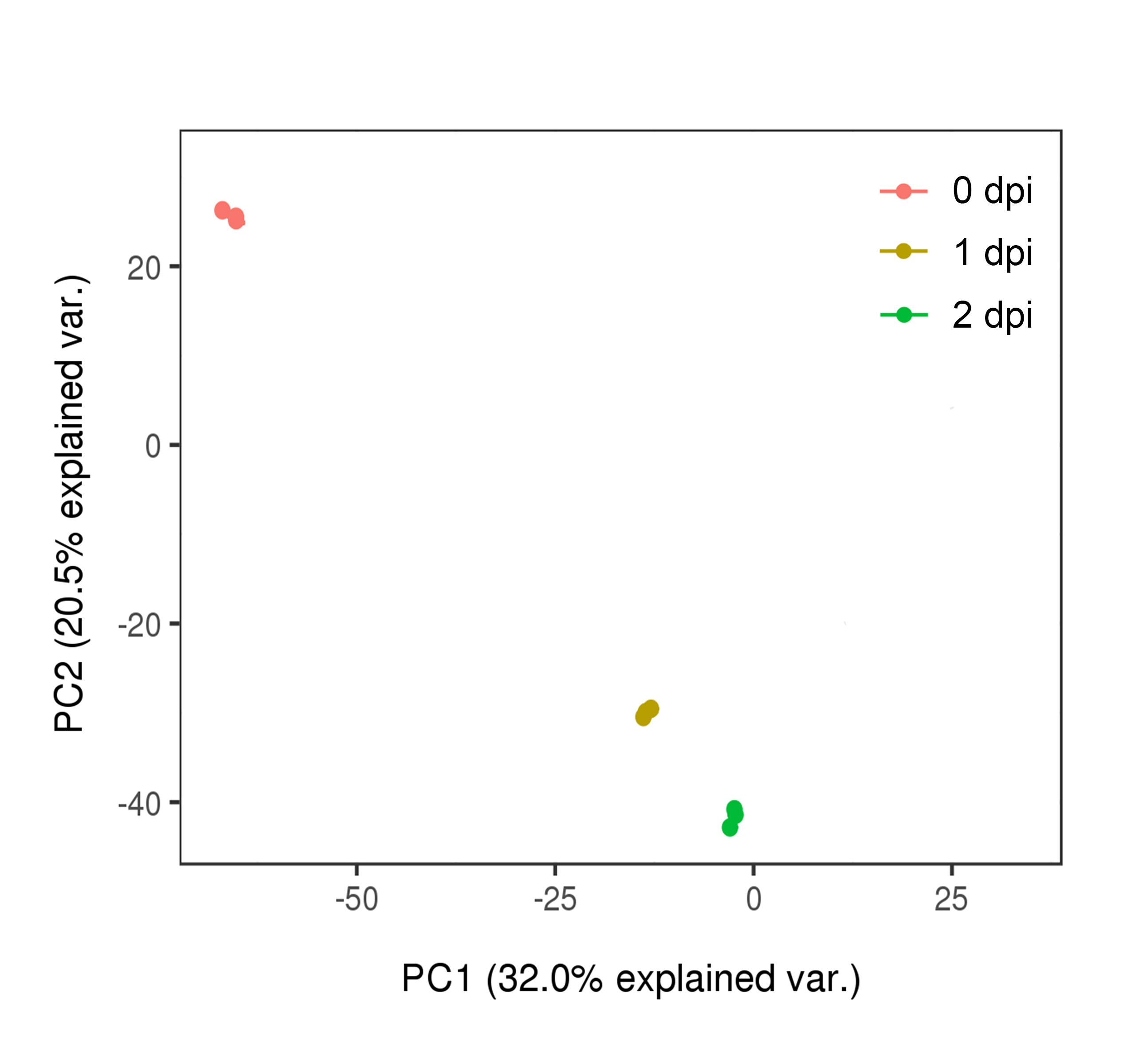

Supplement: Supplementary Figure 1 — Principal component analysis (PCA) of the total proteome data from maize stem at 0, 1, and 2 days after F. graminearum infection. Protein samples at each time point have three replicates. [file Image_1.JPEG]

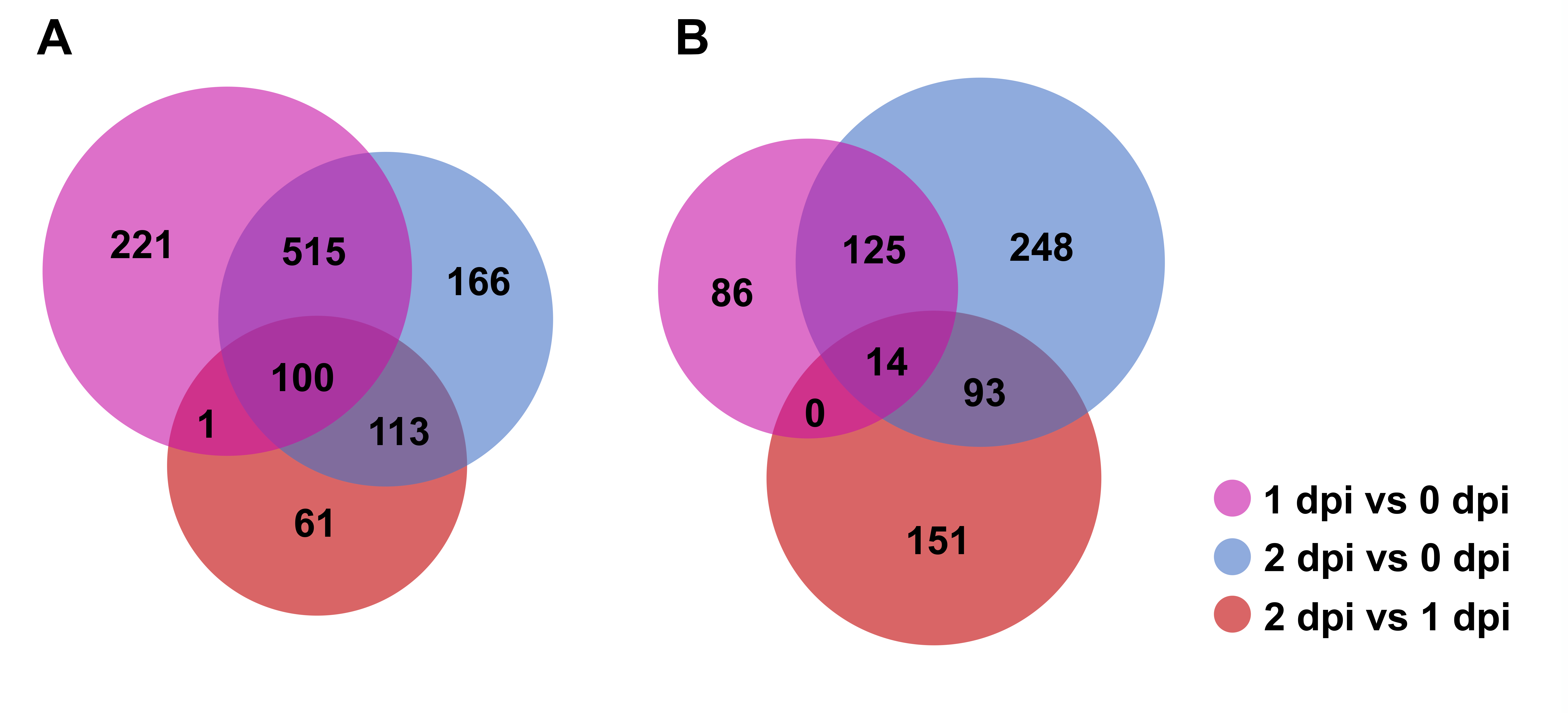

Supplement: Supplementary Figure 2 — Venn diagram analysis of the DEPs that were up- or down-regulated in maize stem at 0, 1, and 2 days post F. graminearum infection. (A) Venn diagram of up-regulated proteins. (B) Venn diagram of down-regulated proteins. [file Image_2.JPEG]

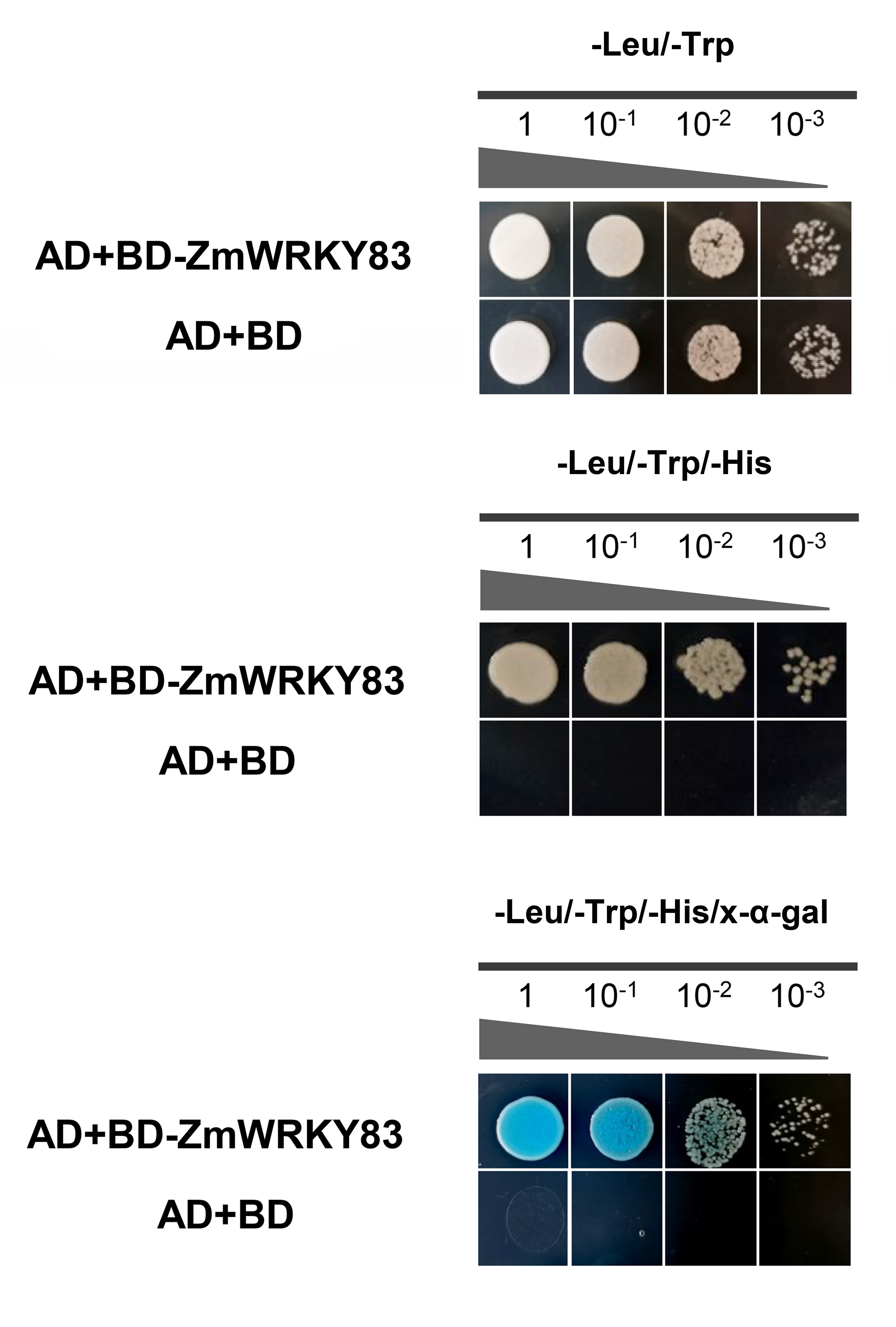

Supplement: Supplementary Figure 3 — Transactivation activity analysis of ZmWRKY83 in yeast. pGBKT7 empty vector (BD) was used as the negative control. The results were determined by the growth of the yeast strains SD/-Leu/-Trp, SD/-Leu/-Trp/-His and SD/-Trp/-His/-Ade/x-α-gal media for 3–5 days at 30°C. [file Image_3.JPEG]

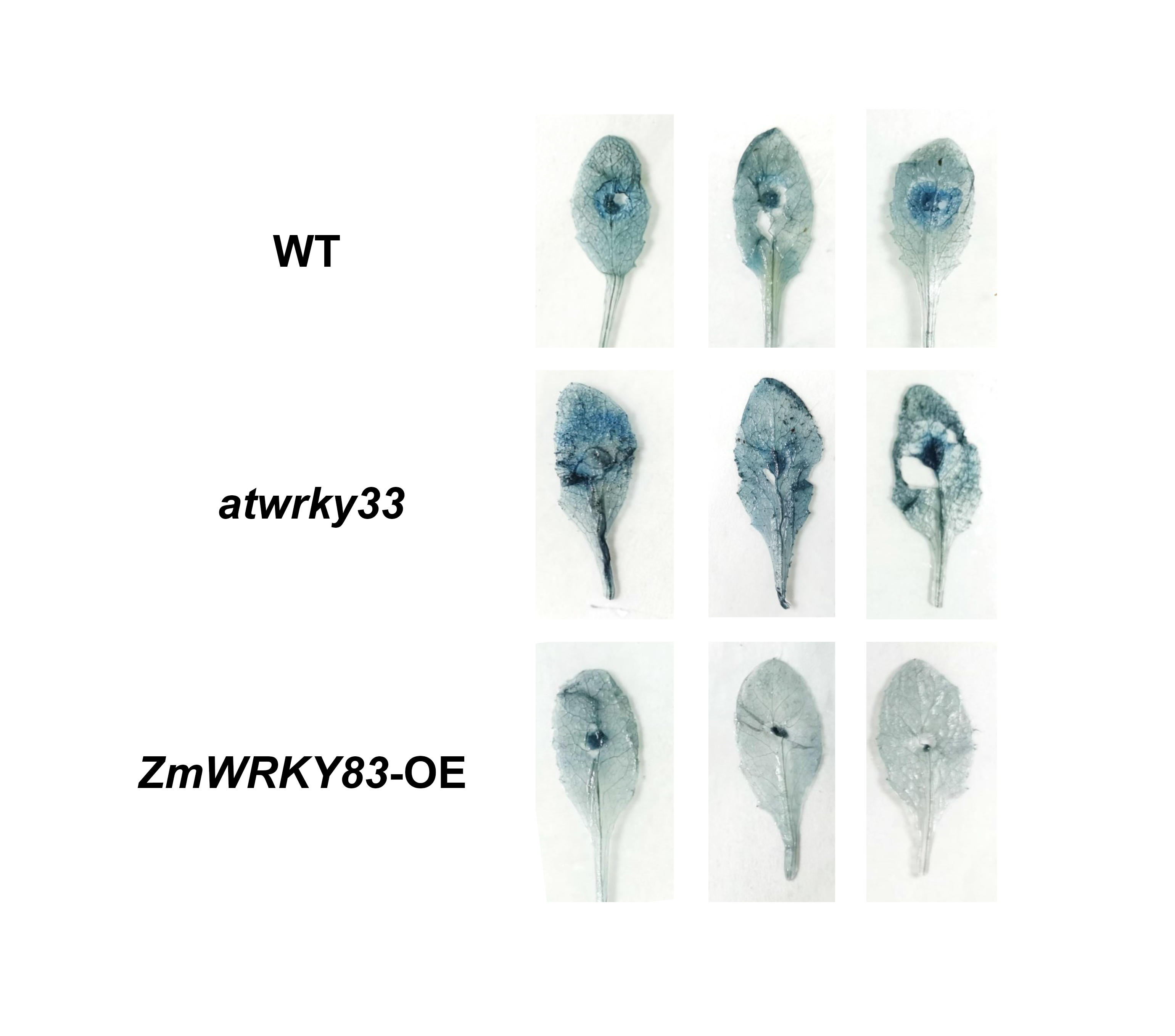

Supplement: Supplementary Figure 4 — Trypan blue staining of leaves from Col-0, atwrky33 mutant, and ZmWRKY83 overexpressing transgenic plants at 7 days after B. cinerea infection showing the extent of death cell triggered by pathogen infection. [file Image_4.JPEG]
